# Supplementary material for: Identification of photoperiod-induced specific miRNAs in the adrenal glands of Sunite sheep (Ovis aries)
Source: Front Vet Sci. 2022 Jul 22;9:888207. doi: 10.3389/fvets.2022.888207 (PMC9354845; doi:10.3389/fvets.2022.888207)
Supplement: Supplementary file 1 [file Data_Sheet_1.ZIP › Supplementary file/miRDeep2 file.zip!/miRDeep2 file/Novel_Structure/SP42A3/Novel_316.pdf]

Provisional ID	:	NC_019464.2_13275
Score total	:	2
Score for star read(s)	:	-1.3
Score for read counts	:	0
Score for mfe	:	1.7
Score for randfold	:	1.6
Score for cons. seed	:	
Total read count	:	101
Mature read count	:	101
Loop read count	:	0
Star read count	:	0

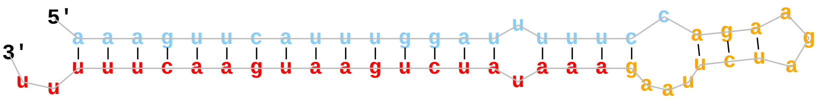

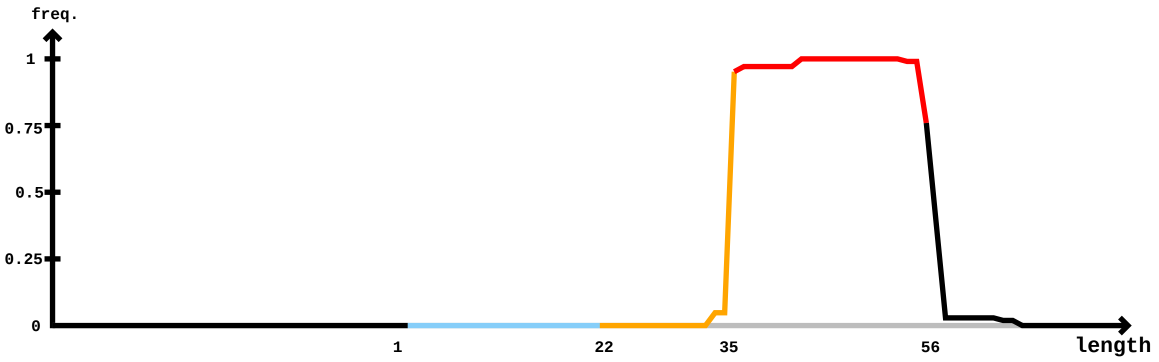

Star   Mature

```
5'- ugucauaaaaggguaaauacuguugaguuggucaaaaaguucauuuggauuuuuccagaagaucuuaagaaauaucugaaugaacuuuuuggccaacccgauuuauaauu   -3'    exp
    .....................((((.((((((((((((((((((((((((.((((.(((...)))...)))).)))))))))))))))))))))))).))))........    reads   mm   sample
    ...................................................................agaaaAaucugaaugaacuuuuu....................    5       1    seq
    .....................................................................aaaAaucugaaugaacuu.......................    1       1    seq
    .....................................................................aaaAaucugaaugaacuuuu.....................    24      1    seq
    .....................................................................aaaAaucugaaugaacuuuuu....................    69      1    seq
    ......................................................................aauaucugaaugaacuuuuu....................    2       0    seq
    ............................................................................ugaaugaacuuuuuggccaac.............    1       0    seq
    ............................................................................ugaaugaacuuuuuggccaaccA...........    1       1    seq
    ............................................................................ugaaugaacuuuuuggccaaccU...........    1       1    seq
```
